# Parents' reported preference scores for childhood atopic dermatitis disease states

**DOI:** 10.1186/1471-2431-4-21

**Published:** 2004-10-18

**Authors:** Joëlle Y Friedman, Shelby D Reed, Kevin P Weinfurt, Kristijan H Kahler, Emmanuel B Walter, Kevin A Schulman

**Affiliations:** 1 Center for Clinical and Genetic Economics, Duke Clinical Research Institute, Duke University Medical Center, PO Box 17969, Durham, NC 27715 USA; 2 Novartis Pharmaceuticals Corporation, East Hanover, NJ 07936 USA; 3 Duke Children's Primary Care, Department of Pediatrics, Box 3675, Duke University Medical Center, Durham, NC 27710 USA

## Abstract

**Background:**

We sought to elicit preference weights from parents for health states corresponding to children with various levels of severity of atopic dermatitis. We also evaluated the hypothesis that parents with children who had been diagnosed with atopic dermatitis would assign different preferences to the health state scenarios compared with parents who did not have a child with atopic dermatitis.

**Methods:**

Subjects were parents of children aged 3 months to 18 years. The sample was derived from the General Panel, Mommies Sub-Panel, and Chronic Illness Sub-Panel of Harris Interactive. Participants rated health scenarios for atopic dermatitis, asthma, and eyeglasses on a visual analog scale, imagining a child was experiencing the described state.

**Results:**

A total of 3539 parents completed the survey. Twenty-nine percent had a child with a history of atopic dermatitis. Mean preference scores for atopic dermatitis were as follows: mild, 91 (95% confidence interval [CI], 90.7 to 91.5); mild/moderate, 84 (95%CI, 83.5 to 84.4); moderate, 73 (95%CI, 72.5 to 73.6); moderate/severe, 61 (95%CI, 60.6 to 61.8); severe, 49 (95% CI, 48.7 to 50.1); asthma, 58 (95%CI, 57.4 to 58.8); and eyeglasses, 87(95%CI, 86.3 to 87.4).

**Conclusions:**

Parents perceive that atopic dermatitis has a negative effect on quality of life that increases with disease severity. Estimates of parents' preferences can provide physicians with insight into the value that parents place on their children's treatment and can be used to evaluate new medical therapies for atopic dermatitis.

## Background

Atopic dermatitis is the most common of childhood skin diseases, with a lifetime prevalence in children of 10% to 20% [1]. The disease is most common in industrialized countries and among Caucasians and Asians [2]. Annual total costs of treatment are estimated to range from $0.9 to 3.8 billion in the United States [3].

Atopic dermatitis can have a negative impact on quality of life by affecting psychosocial adjustment in children. Lapidus and Kerr [4] report that atopic dermatitis can cause embarrassment, disrupt sporting activities in older children, and interfere with employment opportunities among adults. The disease can also have a negative impact on families. Parents report feelings of guilt, exhaustion, frustration, and helplessness [4-7]. Atopic dermatitis can disrupt sleep in patients and their family members, and parents can miss work or avoid outside work altogether to care for a child with the disease [4,8]. Fivenson et al [9] found that 50% of the total burden of illness of atopic dermatitis is associated with lost productivity. Specifically, they found that days lost from work and nights of sleep lost were high among parents of young children with atopic dermatitis [9]. These stresses take additional tolls on familial relationships and are confounded in low-income families, who often have minimal access to social support mechanisms [4].

As the need to control increasing medical expenditures continues to mount, formal economic evaluations are taking on a prominent role in assessing the value of new medical therapies. To properly evaluate the impact of new therapies for atopic dermatitis, patients' health-related quality of life (HRQOL) must be considered. Although a number of quality-of-life evaluations have been conducted for adults and children affected with atopic dermatitis [4,7,9-12], quality-of-life adjustments in cost-utility analyses must be performed using preference weights. Preference weights represent summary measures of HRQOL associated with individual health states and are necessary to calculate quality-adjusted life-years (QALYs) for use in cost-utility analyses.

Although the prevalence of atopic dermatitis is highest in children, the existing literature on patient preferences for atopic dermatitis is limited to the adult population [13,14]. However, eliciting preferences from children may not be possible, because they lack the necessary language skills and cognitive abilities to interpret and respond to questions used to evaluate preferences. Evidence suggests that proxy reports by parents may provide valid estimates of HRQOL in children [15]. Therefore, our primary objective was to elicit preference weights from parents for health states corresponding to children with various levels of severity of atopic dermatitis. In addition, we evaluated the hypothesis that parents with children who had been diagnosed with atopic dermatitis would assign different preferences to the health state scenarios compared with parents who did not have a child with atopic dermatitis.

## Methods

### Preference assessment instrument

Patient preferences can be elicited using standard gamble or time-tradeoff or direct rating methods such as a visual analog scale. Because both the standard gamble and the time-tradeoff exercises involve choices between two alternatives that involve a chance of death or longevity, we believed it was unethical to ask parents to participate in such exercises when children were the subjects. Therefore, our choice for eliciting preferences was the rating scale.

We developed case scenarios for 5 levels of atopic dermatitis severity – mild, mild/moderate, moderate, moderate/severe, and severe. These severity levels were created by combining the characteristics of an Investigator Global Assessment (IGA) and the Eczema Area and Severity Index (EASI) score [16]. Each scenario included descriptions of erythema, infiltration and/or papulation, excoriation, and lichenification, as well as location of body area affected (Table 1). Efforts were made to ensure that the scenarios were descriptive, explicit, nonjudgmental, and targeted to an eighth-grade reading level. A medical artist developed images to depict the descriptions of atopic dermatitis. We also included 2 additional scenarios – one that described wearing glasses and another that described suffering from asthma – to compare the preferences for atopic dermatitis health states to nondermatological health states. A pediatrician and a pediatric dermatologist reviewed the descriptions and illustrations to ensure their validity and realism. The scenarios were revised based on their comments.

**Table 1:** Scenario descriptions

Severity	Scenario
Mild	• The area looks like a light pink or white, dusty rash.
	• It is affecting the cheeks.
	• It is rarely itchy and your child scratches it only a few (about 3) times a day.
	• There are only a few (about 3) slightly bumpy areas.
	• There is no oozing or crusting.
	• The skin is not dry or leathery.
	• Sleep is rarely disrupted by itching.
Mild/Moderate	• The area looks like a light pink or white, dusty rash.
	• It is affecting the cheeks and the chin.
	• It is somewhat itchy and your child scratches it about 5 times a day.
	• There are about 5 slightly bumpy areas.
	• There is no oozing or crusting.
	• The skin is not dry or leathery.
	• Sleep is somewhat disrupted by itching. Your child loses about 15 minutes of sleep each night because of scratching.
Moderate	• The area looks like a dark pink rash.
	• It is affecting the cheeks, the chin and the inside of the elbows.
	• It is moderately itchy and your child scratches it often (about 15 times) during the day.
	• There are about 7 moderately bumpy areas.
	• There is no oozing or crusting.
	• The skin is not dry or leathery.
	• Sleep is disrupted by itching. Your child loses about 1 hour of sleep each night because of scratching.
Moderate/Severe	• The area looks like a dark pink rash.
	• It is affecting the cheeks, the chin and the inside of the elbows, and the back of the knees.
	• It is itchy and your child scratches it often (about 30 times) during the day.
	• There are about 10 moderately bumpy areas.
	• There is some light oozing or crusting in one area.
	• The skin is not dry or leathery.
	• Sleep is disrupted by itching. Your child loses about 2 hours of sleep each night because of scratching.
Severe	• The area looks like a red rash.
	• It is affecting the cheeks, the chin and the inside of the elbows, and the back of the knees, and the trunk of the body.
	• It is very itchy and your child scratches it scratching continuously throughout the day.
	• There are numerous bumpy areas.
	• There is oozing or crusting in some areas.
	• The skin is dry and leathery in some areas.
	• Sleep is disrupted by itching. Your child loses about 3 hours of sleep each night because of scratching.

Using cognitive interview techniques, we pilot-tested the preference assessment instrument in a convenience sample of 20 parents of children who were being evaluated at a local children's primary care clinic to assess patients' understanding of the instrument and its instructions. The instrument was further revised based on the results of the pilot test.

### Survey administration

A health state preference assessment asks subjects to make judgments regarding the value of particular health states [9]. Preferences for health states can be elicited from patients with disease (or their family members), from patients at risk for disease, or from the general public [17,18]. We elected to develop preferences from family members of children who currently had atopic dermatitis or were at risk for the disease. To obtain responses from a broad range of respondents in an efficient manner, we recruited participants over the Internet. The sample was derived from the General Panel, the Mommies Sub-Panel, and the Chronic Illness Sub-Panel of Harris Interactive (Rochester, NY), an international market research and consulting firm. The General Panel is a multimillion-member panel of respondents who register to participate in The Harris Poll online panel. The Mommies Sub-Panel is a subpanel of respondents with children aged up to 2 years. The Chronic Illness Sub-Panel identifies respondents (or household members) who have been diagnosed with at least 1 of more than 44 chronic medical conditions, including skin conditions. (The Mommies and Chronic Illness Sub-Panels are part of The Harris Poll online panel. Aside from parental and health status, their members do not differ systematically from members of the General Panel.).

Subjects were invited to participate in the survey from February 12 through 14, 2002, and were asked to register at a specific survey site. After consent was obtained online, subjects completed the survey. Respondents were offered the incentive of a chance to win one of five $100 prizes. Respondents had to be adults with children aged 3 months to 18 years in order to be included in the study. The study was approved by the institutional review board of Duke University Medical Center.

Clinical data were based on self-report and included information on diagnosis history and severity level. Specifically, respondents were asked if they had a child between the ages of 3 months and 18 years who had ever been diagnosed by a medical professional with atopic dermatitis. If they responded "yes," they were then asked to describe the child's atopic dermatitis at its worst point by selecting from the following response options: mild, mild/moderate, moderate, moderate/severe, severe.

For the preference assessment, each respondent was given the scenarios in the same order – mild through severe atopic dermatitis, asthma, and glasses. Subjects were instructed to indicate on a scale ranging from 100 (perfect health) to 0 (death) how good or bad they believed it would be to be a child experiencing the scenario depicted. Respondents recorded their values using a movable pointer on the scale. Respondents whose Internet browser software did not support the movable pointer entered their numerical responses manually into a required field. All 7 values were summarized at the end of the survey to allow respondents to review and, if desired, revise their ratings.

### Data analysis

Descriptive statistics were used to describe the sample. Because subjects provided responses across severity levels, a repeated-measures analysis of variance was conducted using polynomial contrasts for the within-subjects (severity) effect. A *P *value of ≤ .05 was used as the criterion for statistical significance.

## Results

Of the 28105 subjects contacted, 6131 (22%) responded. Of the 6131 respondents, 3539 (57.7%) met the inclusion criteria, consented to participate in the study, and completed the survey (Table 2). The mean age was 41 years; 93% of the subjects were women; and 90% were white. Nonresponders were similar to responders with respect to age, sex, and race/ethnicity. More than 98% of the sample had at least a high school education, with 83% of respondents completing at least some college courses. Overall, the sample reflected moderate- to high-income families, with 74% having an annual household income of at least $35000. Thirty percent of the parents had a child with atopic dermatitis, 55% had a child with asthma, and 46% had a child who wore glasses.

**Table 2:** Subject characteristics

Characteristic	Responders (n = 3539)	Nonresponders (n = 21974)
Age		
Mean (SD)	40.6 (6.2)	41.0 (6.4)
Range	18–64	19–76
Female sex	3298 (93.2)	20263 (92.2)
Race/ethnicity*		
White	3045 (89.7)	18507 (84.2)
Black/African-American	136 (4.0)	1098 (5.0)
Hispanic	79 (2.3)	703 (3.2)
Asian/Pacific Islander	21 (0.6)	171 (0.8)
Native American	38 (1.1)	316 (1.4)
Mixed/other	77 (2.3)	520 (2.4)
Unknown	0	559 (2.5)
Declined to answer	0	100 (0.5)
Education level†		
Did not complete high school	55 (1.6)	706 (3.2)
High school degree	555 (15.7)	4366 (19.9)
Some college	1391 (39.3)	9074 (41.3)
College degree	991 (28.0)	5179 (23.6)
Some graduate school or degree	543 (15.4)	2568 (11.7)
Unknown	0	81 (0.4)
Annual household income‡		
<$15000	107 (3.5)	868 (3.9)
$15000$24999	262 (8.6)	1983 (9.0)
$25000–$34999	410 (13.4)	2945 (13.4)
$35000–$49999	665 (21.7)	4177 (19.0)
$50000–$74999	806 (26.3)	5065 (23.0)
$75000–$99999	406 (13.3)	2003 (9.1)
$100000–$149999	220 (7.2)	1442 (6.6)
$150000–$199999	85 (2.8)	273 (1.2)
$200000–$249999	61 (2.0)	117 (0.5)
≥ $250000	16 (0.5)	103 (0.5)
Declined to answer	24 (0.8)	1 (0.0)
Unknown	0	2997 (13.6)
Employment status §		
Employed full-time	1766 (80.0)	9070 (42.2)
Employed part-time	554 (15.7)	2355 (11.0)
Self-employed	281 (7.9)	1588 (7.4)
Not employed but looking for work	119 (3.4)	852 (4.0)
Not employed and not looking for work	70 (2.0)	588 (2.7)
Retired	40 (1.1)	258 (1.2)
Student	149 (4.2)	3071 (14.3)
Homemaker	974 (27.5)	3566 (16.6)
Disabled	0	138 (0.6)
Not sure	4 (0.1)	0
Declined to answer	4 (0.1)	0
Not applicable	0	0
Number of children in household		
Mean (SD)	1.9 (1.0)	1.9 (1.2)
Range	0–9	0–15
Country of residence		
Australia	2 (0.1)	--
Canada	2 (0.1)	--
United States	3535 (99.9)	--

Values are reported as number (percentage) unless otherwise indicated.

*There were 143 missing responses for this variable.

† There were 4 missing responses for this variable.

‡ There were 477 missing responses for this variable.

§ Responses sum to more than 100% because respondents could select more than one answer. Nonresponders did not have this option. There were 488 missing responses for this variable.

Table 3 displays the characteristics of the children's atopic dermatitis (n = 1017). Seventy-eight percent (n = 806) of children were diagnosed more than a year ago. Sixteen percent (n = 160) of the sample described their child's atopic dermatitis as mild and 8% (n = 78) described their child's atopic dermatitis as severe. Thirty-five percent (n = 357) of the sample reported their child's atopic dermatitis under limited control or uncontrolled.

**Table 3:** Disease characteristics for children with atopic dermatitis

Characteristic	Subjects (n = 1017)
Time of diagnosis	
≤ 1 month ago	119 (11.7)
7 months to <12 months ago	95 (9.3)
1 year to 5 years ago	467 (45.9)
> 5 years ago	336 (33.0)
Disease severity	
Mild	160 (15.7)
Mild to Moderate	236 (23.2)
Moderate	277 (27.2)
Moderate to Severe	266 (26.2)
Severe	78 (7.7)
How well controlled?	
Complete	206 (20.3)
Good control	409 (40.2)
Limited control	330 (32.4)
Uncontrolled	27 (2.6)
No treatment	45 (4.4)

Values are reported as number (percentage) unless otherwise indicated.

The mean values for all participants are presented in Table 4. Among the atopic dermatitis health states, there was a progressive decline in respondents' preferences, with the mildest state receiving the highest mean preference score and the severe state receiving the lowest mean preference score. On average, preferences for asthma were higher than for severe atopic dermatitis but lower than moderate/severe atopic dermatitis. Not surprisingly, wearing glasses received a higher preference value than suffering from asthma. Average preference values for the glasses health state were ranked between the mild and mild/moderate atopic dermatitis health states.

**Table 4:** Average health state preference values

Health State	Mean	Median	95% Confidence Interval
Mild atopic dermatitis	91.1	95.0	90.7–91.5
Mild/moderate atopic dermatitis	83.9	88.0	83.5–84.4
Moderate atopic dermatitis	73.1	76.0	72.5–73.6
Moderate/severe atopic dermatitis	61.2	63.0	60.6–61.8
Severe atopic dermatitis	49.4	50.0	48.7–50.1
Asthma	58.1	60.0	57.4–58.8
Wearing eyeglasses	86.8	94.0	86.3–87.4

**Figure 1:**
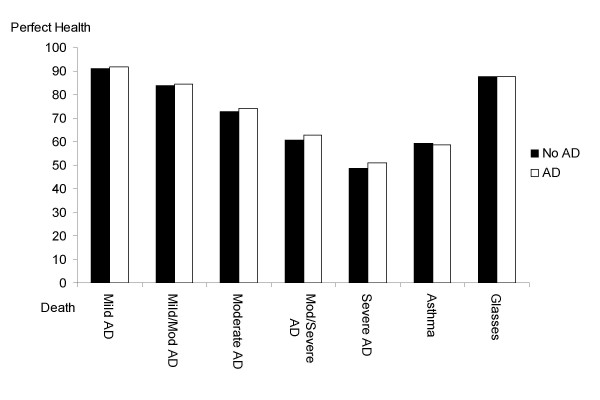
Comparison of overall ratings, stratified by children with atopic dermatitis and children without atopic dermatitis

There was a significant effect of severity (F_4,3391 _= 3065.66; *P *= .0001). The linear effect of severity (F_1,3394 _= 11454.90; *P *< .0001) indicated that preference ratings significantly decreased as the severity of the health states increased (Figure 1). Furthermore, there was a significant main effect for preferences reported by parents of children with atopic dermatitis as compared to parents of children without atopic dermatitis (F_1,3394 _= 8.10; *P *= .0045). Across all health states, parents of children with atopic dermatitis gave a slightly higher mean preference (72.85 [SD, 13.50]) compared to parents whose children did not have atopic dermatitis (71.34 [SD, 14.19]) (Figure 1).

There was no significant severity by parent group interaction (F_4,3391 _= 1.21; *P *= .31), indicating that the differences across health states were the same for both parent groups.

## Discussion

Our study evaluated preferences for 5 health states for atopic dermatitis. The aggregate values for each health state may be used for computing QALYs for new therapies that treat atopic dermatitis or can be used to help physicians make more informed decisions by considering parents' perceptions of atopic dermatitis measured on a continuum from perfect health to death. The differences in average values across the 5 health states were generally consistent, with mild at 91, mild/moderate at 84, moderate at 73, moderate/severe at 61, and severe at 49.

Lundberg and colleagues [13] found that the mean health-state utility using a rating scale for patients with atopic dermatitis was 77, a value only slightly higher than our average health-state utility of 73. This small discrepancy could be explained by several factors. First, Lundberg et al [13] asked adult patients to provide ratings for themselves, whereas our study asked parents to provide ratings for children. Parents might feel that a given health state is worse for their children than it would be for themselves. Second, Lundberg et al [13] asked patients to provide a rating for their current health state, whereas our study asked parents to assign utilities to 5 varying levels of severity of atopic dermatitis. The average severity level of Lundberg et al's [13] sample might have been slightly lower than the average severity among our 5 health states, making the mean preference value slightly higher. By obtaining preferences for varying levels of severity, our results have greater applicability in various types of models for decision making.

Although preference ratings were systematically higher among parents of children with atopic dermatitis than among parents whose children did not have atopic dermatitis, the magnitude of the difference was small (difference = 1.5). Research on adults who rate the health states of other adults has suggested that people who have experienced a particular health state are more likely to assign a higher value to it than others who are asked to imagine the health state [19-21]. The lack of a larger difference between the parent groups in our study could indicate that parents evaluate health states of children the same, regardless of whose children they are, because of a general concern for all children. Regardless of the reason, these results have important implications for the use of community-based preference weights, as opposed to patient-based (or parent-based) weights, in preference-weighted decision analyses. For this limited therapeutic domain, our study shows that parents in the general community would supply approximately the same preferences as parents whose children suffer the condition under study. Future research should consider whether this consistency holds for other serious childhood diseases, such as pediatric cancer.

Our study has several limitations. First, by using the Internet, respondents did not have an opportunity to ask questions if they did not understand what they were being asked to do. However, we feel that this limitation was negligible, because the instrument was pilot-tested using in-person interviews, and the very large majority of responses were ranked appropriately (eg, mild ranked higher than severe). Secondly, there may be a sample bias in using the Harris Interactive database. Our sample reflected a predominately white, female cohort from a high socioeconomic class and may present generalizability issues. However, we received responses from 351 (14%) nonwhite respondents and 779 (22%) responses from participants with an annual household income of less than $35000, providing a sufficient number of responses to test for differences by race/ethnicity and income level. Further research will be needed to corroborate our findings using a population-based sample. Third, since these data are self-reported, some parents may have misclassified their children as having or not having atopic dermatitis, potentially biasing our results. Fourth, it is unclear whether physician assessment of severity would correspond with the severity levels that we assigned to the health states. However, physicians could evaluate the descriptions provided to judge whether their assessments are consistent. Finally, people enrolled in the Harris Interactive database are computer users who may be more motivated to participate in a survey than the general population. It is unclear how or in what direction these sample biases might affect the results of our analyses.

We had an apparent response rate of 22%. Harris Interactive generally achieves a 15% to 20% response rate when using the Chronic Illness Sub-Panel. While our response rate was higher than Harris' average response rate, possible reasons for why the response was low could be attributed to the nature of the study design. First, because subjects were contacted by e-mail, it is possible that some subjects did not open the e-mail message until after the survey deadline. Second, Harris Interactive panel members agree to be notified about survey opportunities, but do not agree to participate in each survey. Since the characteristics of responders and nonresponders did not differ, we have no reason to believe that nonresponse bias is exerting a substantial influence on our results.

## Conclusions

The results of this analysis clarify the values that parents of children with atopic dermatitis assign to different atopic dermatitis health states. These assigned values, relative to the comparison states, clearly demonstrate the perceived burden of atopic dermatitis by parents of children suffering from the disease. Understanding the preferences for atopic dermatitis can provide physicians insight into the value that parents place on treatments for their child's disease and in evaluating the cost-effectiveness of therapies for atopic dermatitis.

## Competing interests

This study was supported by a research agreement between Duke University Medical Center and the Novartis Pharmaceuticals Corporation, East Hanover, NJ, which manufactures a cytokine inhibitor for the treatment of atopic dermatitis.

KPW and KAS have received monetary compensation for consultancies, and EBW and KAS have received research grants, from Novartis. KHK is an employee of Novartis.

## Authors' contributions

JYF conceived of and designed the study, performed the statistical analysis, interpreted the data, and drafted the manuscript. SDR conceived of and designed the study and assisted in interpretation of the data and drafting of the manuscript. KPW assisted in interpretation of the data and drafting of the manuscript. EBW and KHK conceived of and designed the study and assisted in drafting of the manuscript. KAS conceived of and designed the study, assisted in drafting of the manuscript, and obtained funding. All authors read and approved the final manuscript.

## Pre-publication history

The pre-publication history for this paper can be accessed here:


